# Relationships among supervisor support, autonomy, job satisfaction and emotional labor on nurses within the Turkey context of healthcare services

**DOI:** 10.3389/fpsyg.2023.1303170

**Published:** 2024-01-30

**Authors:** Sabiha Sevinç Altaş, Hülya Gündüz Çekmecelioğlu, Gönül Konakay, Murat Günsel

**Affiliations:** ^1^Vocational School of Health Services, Sakarya University, Sakarya, Türkiye; ^2^Faculty of Business Administration, Kocaeli University, Kocaeli, Türkiye; ^3^Hereke Omer Ismet Uzunyol Vocational School, Kocaeli University, Kocaeli, Türkiye; ^4^Business Management Ph.D. Program, Beykent University, İstanbul, Türkiye

**Keywords:** supervisor support, autonomy, job satisfaction, emotional labor, nurse occupation

## Abstract

**Introduction:**

Healthcare professionals face the challenging task of regulating their emotions within the workplace, which can lead to significant pressure and stress. For nurses, who work in particularly demanding environments, fulfilling the expectations of emotional labor can be challenging.

**Methods:**

This study explores how nurses’ perceptions of supervisor support and job autonomy can positively influence emotional labor and job satisfaction via Partial Least Squares Structural Equation Modeling (PLS-SEM) technique.

**Results and discussion:**

Job autonomy is found to negatively affect emotional labor but positively impact job satisfaction. Additionally, job satisfaction is a significant precursor to both surface and deep-acting dimensions of emotional labor. Furthermore, job satisfaction mediates the relationship between supervisor support and deep-acting emotional labor, as well as between job autonomy and both surface and deep-acting emotional labor. These findings shed light on the complex dynamics of emotional labor and job satisfaction in healthcare settings.

## 1 Introduction

Studies have consistently indicated that healthcare professionals, particularly nurses, face a myriad of challenges in their work environments, which often require them to manage their emotions effectively. This expectation of emotional labor, coupled with the inherent stressors of the healthcare industry, places significant pressure on nurses and can impact their job satisfaction and, consequently, the overall quality of healthcare services provided (Aiken et al., 2009; Yun et al., 2010; Wu et al., 2018). Italian nurses, for instance, grapple with work–family conflicts and long-term exposure to emotional labor, resulting in increased work-related stress, decreased health, diminished quality of life, and elevated burnout rates (Cortese et al., 2010; Zaghini et al., 2020). Similarly, Swedish nurses experience enhanced job satisfaction when they perceive a positive organizational climate and supportive leadership behaviors (Wallin A. O. et al., 2012).

In the context of Turkish healthcare, nurses confront these same challenges along with issues such as low wage satisfaction and the burden of high patient-to-nurse ratios. Despite the implementation of the “Health Transformation Program” in 2003 aimed at improving the healthcare system in Turkey, there has been limited growth in the number of healthcare personnel per patient. The healthcare workforce in Turkey remains understaffed, with only 217 physicians and 343 nurses and midwives per 100,000 people, significantly below the OECD averages (T.C. Sağlık Bakanlığı Sağlık İstatistikleri Yıllığı, 2021). These issues stemming from the working conditions of nurses not only diminish their work commitment, performance, and organizational efficiency but also have adverse effects on the quality of patient care (Bakker and Demerouti, 2008; Cortese et al., 2013; Zaghini et al., 2020).

Considering these challenges, nurses working in such demanding conditions may find it challenging to exhibit emotional labor toward.

patients, colleagues, and other stakeholders they interact with. However, the perception of a positive work environment can significantly impact nurses’ job satisfaction and their ability to engage in emotional labor. Job satisfaction is a recognized factor that influences positive work attitudes and organizational commitment in both the emotional management and work climate literature (Ashforth and Humphrey, 1993; Morris and Feldman, 1996; Grandey, 2000; Brotheridge and Grandey, 2002; Çekmecelioğlu, 2005; Luthans, 2011; Robbins et al., 2015).

Despite the growing interest in emotional labor and its implications for service quality, there is limited research on the relationships between employees’ perceptions of the work environment, work attitudes such as emotional labor and job satisfaction, especially in the healthcare sector. Consequently, this study comprehensively examines these relationships by focusing on key dimensions of the work environment, namely supervisor support and job autonomy, and their impact on emotional labor and job satisfaction. Moreover, the study investigates the mediating role of job satisfaction in the relationships between supervisor support and emotional labor, as well as between job autonomy and emotional labor.

This study also explores the multifaceted nature of emotional labor, which involves employees managing their emotions in line with organizational expectations. Hochschild (1983) defined emotional labor as the effort individuals exert to display specific emotions required by their jobs or to manage their emotions effectively. Emotional labor requires individuals to adhere to certain emotional display rules, regardless of their genuine emotional state (Ekman, 1973; Hochschild, 1983). Researchers have categorized emotional labor strategies into surface acting (displaying false emotions or suppressing genuine emotions) and deep acting (authentically experiencing and expressing emotions that align with organizational expectations; Brotheridge and Lee, 2002; Grandey, 2003). While these two dimensions are well-studied, there is limited research on the natural-sincere behavior strategy, which entails genuinely displaying emotions without resorting to pretense (Ashforth and Humphrey, 1993; Morris and Feldman, 1996; Grandey, 2000; Brotheridge and Grandey, 2002).

Existing literature has predominantly explored the relationship between emotional labor and work-related outcomes such as burnout, job satisfaction, and turnover intentions (Steinberg and Figart, 1999; Gursoy et al., 2011; Lee and Madera, 2019). While surface acting has consistently been associated with negative work attitudes, studies have shown mixed results regarding the impact of deep acting on these outcomes (Grandey, 2000; Brotheridge and Lee, 2003; Seçer, 2005; Yao et al., 2015; Yin, 2016; Karakaş and Gökmen, 2018). Employees in customer-facing roles, who frequently employ emotion regulation strategies and lack job autonomy, are more susceptible to burnout, whereas those with higher job autonomy can better manage their emotions (Hochschild, 1979; Grandey, 2015). Within the work environment, managerial support and job autonomy are considered essential job resources that contribute to employees’ well-being and positive emotions, as proposed by the Job Demands-Resources Model (JD-R Model; Demerouti et al., 2001; Bakker and Demerouti, 2007).

The JD-R Model, which examines the balance between job demands and job resources and their effects on employee well-being and job stress in healthcare settings, suggests that job demands, such as emotional demands, create tension and pressure on individuals, resulting in negative outcomes when excessive (Demerouti et al., 2001). Conversely, job resources, including organizational and social factors that facilitate employees’ pursuit of work and organizational goals, can mitigate this pressure, enhance well-being, and boost motivation. Maintaining a balance between job demands and resources can lead to positive outcomes such as increased productivity, reduced absenteeism, and improved working relationships. However, an imbalance between these factors can result in reduced focus, increased errors, and higher absenteeism, eventually leading to burnout (Demerouti et al., 2001).

Considering the JD-R Model, this study examines the impact of two critical job resources, supervisor support and job autonomy, on emotional labor and job satisfaction. It further explores the direct influence of job satisfaction on the surface and deep acting dimensions of emotional labor and assesses the mediating role of job satisfaction in the relationships between supervisor support, job autonomy, and emotional labor. By investigating these relationships in the context of Turkish healthcare, this study aims to provide valuable insights into the emotional labor experienced by nurses and its effects on their job satisfaction. Understanding these dynamics is vital for healthcare organizations seeking to enhance the well-being of their nursing staff and improve overall hospital performance.

## 2 Theoretical background and hypothesis development

### 2.1 Emotional labor, supervisor support

As Hochschild stated in his book *“The Managed Heart: Commercialization of Human Feeling”* (1983), in which he discusses the role of emotions in organizational life, employees’ emotions and emotional displays have become commercialized and come under the control of organizations in order to increase the quality of interactions with customers. Moreover, the presentation of desired emotions determined by organizations has become a key component of the work performed by many employees (Morris and Feldman, 1996, 967). Emotional labor is defined as *“the work of making an effort to feel the right emotions required by the job, or the management of feelings”* (Hochschild, 1983). Similarly, emotional labor is defined by Morris and Feldman (1996) as “the effort, plan and control of individuals to express the emotions expected by the organization in the process of interpersonal interaction”; It is defined by Ashforth and Humphrey (1993) as *“the set of behaviors shown to express the appropriate emotional state.”* Although there is an opinion that the issue of emotional labor is more important in jobs in the service sector that require face-to-face relationship with customers (Hochschild, 1983; Wharton, 1999; Sutton and Wheatley, 2003), it is seen that the individual is expected to show emotional labor for all parties with whom s/he communicates in all sectors and working environments (Akgün et al., 2011; Akturan et al., 2017; Mucun et al., 2021).

As stated in the literature on regulation of emotions (Ashforth and Humphrey, 1993; Morris and Feldman, 1996), the environment is seen as a very important factor in understanding emotional management. In this context, the work environment or organizational climate has a feature that determines the type and level of emotional labor of employees. Management support or supervisor support, which is one of the most important dimensions of the organizational climate, includes behaviors where employees’ ideas are taken in decision-making and problem solving and included in business processes, giving importance to employees rather than a controlling management approach and providing feedback about the work. It has been found that this management style instills a sense of trust in the person and enables the individual to produce more creative solutions by arousing the feeling of being in control of his/her job (Amabile et al., 1996; Brown and Leigh, 1996; Cummings and Oldham, 1997; Gümüşlüoğlu and Ilsev, 2009). It is stated that supervisor support will increase the appropriate emotional display by ensuring that the emotions to be displayed by the employees are seen as a requirement of the job role. In this process, the clarity of the organizational rules for the emotional display will increase the skills of the employees and the training process of the employees, and the display of appropriate emotions for the job (Morris and Feldman, 1996). A supportive work environment enables the person to feel better and cope with stress in jobs that require close communication with the customer, allowing the organization to display the emotions expected by the organization and making the person exert less emotional labor (Grandey, 2000; Grandey et al., 2004). Some of the factors that determine the state of employees showing desired emotions in the workplace and transforming these emotions into behaviors are the determination of norms on how to express emotions in the workplace, the clarity of emotion display rules and the use of socialization mechanism to learn them (Rafaeli and Sutton, 1987; Rafaeli and Sutton, 1990). The support of the management is related to the leadership characteristics and behaviors of the managers in the organizational environment and to support their subordinates by using these characteristics. According to the manager-employee exchange theory, an exchange occurs between the leaders and the subordinates, who take care of the employees individually by providing trust, and in response to the trust and interest given by the leader, the subordinates feel obliged to regulate their emotions and behave more sincerely (Erdoğan et al., 2004, 395). A supportive, friendly and collaborative organizational climate reduces the suppression of emotions and negative factors such as emotional disharmony (Ryu et al., 2020, 486). Abraham (1998) suggested that the social support displayed by the manager, co-worker, and the organization in the workplace can reduce the harmful effects of stress caused by emotional labor. It has been determined that perceived supervisor support in tourism businesses positively affects emotional labor (Chen et al., 2012) while perceived organizational support has been found to act as a mediator between emotional labor and various business outcomes in retail service firms (Duke et al., 2009) and a mobile phone company (Nixon et al., 2011). Similarly, in a study conducted on Korean airlines employees, it was found that managerial support played a moderator role between deep acting and job performance (Kim et al., 2017). In a study conducted with tourism sector employees, it was found that there is a positive relationship between the support provided by managers and colleagues and the deep acting, and there is no significant relationship between surface acting and social support (Xu et al., 2020). Studies on emotional labor and leadership styles in Turkey show that the supportive behaviors of managers with various leadership styles toward their subordinates significantly affect emotional labor behavior (Beğenirbaş and Yalçın, 2012; Cinnioğlu and Salha, 2017; Kafadar and Kaygın, 2017; Kavak and Kaygın, 2018). Therefore, in this study, it is thought that the supportive and motivating behaviors of the supervisors will affect the surface acting behaviors of the employees negatively and the deep acting behaviors positively, and the following hypotheses are developed:

*H*1: Supervisor support negatively affects surface acting dimension of emotional labor.

*H*2: Supervisor support positively affects deep acting dimension of emotional labor.

### 2.2 Supervisor support and job satisfaction

Management support is an element that shapes the perceptions of the individual regarding the organizational climate and enables them to perceive the climate positively (Grandey, 2000). In this process, it is seen that especially the first supervisors of the employees play a very important role in the development of positive work attitudes of the employees. A supportive, friendly and collaborative organizational climate reduces the suppression of emotions and negative factors such as emotional disharmony, increases employee job satisfaction and team performance, and reduces the intention to leave (Grandey, 2000; Ryu et al., 2020, 486). Similarly, it is seen that employees develop positive job attitudes and behaviors in an organizational climate where management approach supports participation and employees participate in decision-making processes (Robbins et al., 2018). Supervisors enable employees to develop creative attitudes when they set clear goals, communicate with their employees, and support their employees’ ideas (Amabile et al., 1996). Morris and Feldman (1996, 1005) also state that when employees receive social support from others in the work environment, employees will help others in coping with stress, thus increasing the individual’s job satisfaction level and work efficiency. Therefore, the following hypothesis has been developed:

*H*3: Supervisor support positively affects job satisfaction.

### 2.3 Emotional labor and job satisfaction

Research on emotional labor has yielded conflicting results regarding the relationship between adhering to emotional display rules and job satisfaction. Hochschild (1983) highlighted the negative consequences of managing emotions, including increased job stress, burnout, absenteeism, physical ailments like headaches, and reduced job satisfaction. Studies in health psychology (Dembroski et al., 1985; Gross, 1998) have linked the suppression of negative emotions to physical symptoms such as high blood pressure and heightened stress. Consequently, it is plausible that the regulation and suppression of emotions may have an adverse association with job satisfaction (Diefendorff and Richard, 2003).

In contrast, Brotheridge and Grandey (2002) found that perceiving demands for positive emotional expression positively correlated with feelings of personal accomplishment, potentially resulting in more favorable job attitudes (Ashforth and Humphrey, 1993). Accordingly, individuals who perceive the need to display positive emotions may seek personal benefits by genuinely experiencing positive emotions (Grandey, 2000). Similarly, Schaubroeck and Jones (2000) suggested that positive emotional displays could enhance job satisfaction by facilitating the experience of positive emotions.

Notably, a recent study (Xu et al., 2020) determined that deep acting positively correlated with attitudinal variables such as job satisfaction and organizational commitment, while surface behavior showed no significant relationship with job satisfaction.

This study delves into whether job satisfaction acts as a determinant of emotional labor. While prior research has explored whether emotional labor leads to job satisfaction, this study posits that job satisfaction could also influence emotional labor. An employee content with their job and its characteristics might perceive less stress in the emotional regulation process and exhibit more deep acting. This perspective aligns with the Job Demands-Resources (JD-R) model proposed by Demerouti et al. (2001), which categorizes job factors into demands and resources. Job demands involve aspects that require sustained effort or skill, potentially resulting in physiological and psychological costs like job pressure and emotional demands. Conversely, job resources can aid in achieving work-related objectives while reducing associated physiological and psychological costs, promoting personal growth. Such resources encompass career opportunities, supervisor guidance, role clarity, and autonomy.

The JD-R Model posits that a balance between job demands and resources enhances well-being, motivation, and productivity, whereas an imbalance leads to increased job stress and burnout (Demerouti et al., 2001). Consequently, individuals satisfied with their job and its conditions may engage in less surface acting and more deep acting. In this context, job satisfaction may emerge as a determinant of emotional labor. Grounded in these theoretical frameworks and research findings, the study formulates the following hypotheses:

*H*4: Job satisfaction negatively affects surface acting dimension of emotional labor.

*H*5: Job satisfaction positively affects deep acting dimension of emotional labor.

Schneıder and Snyder (1975, 31) defines job satisfaction as a personal assessment of the job and work environment (the job itself, management’s attitude), or the results of the job (wages, job security), or the employees’ perception of the job and what it offers, and their emotional response to this perception (Luthans, 2011, 114). These definitions make it apparent that one’s perceptions of and satisfaction with one’s job and working conditions determine job satisfaction. As a result, the person compares the resources at his or her disposal with what the organization expects of them. Job satisfaction is derived from the harmony between these two factors. The JD-R model suggests that achieving a balance between job demands and resources might boost employee job satisfaction. The most crucial factor for enabling employees to have a favorable perception of their work environment is supervisor support. Positive work attitudes such as job satisfaction and organizational commitment are increased by supportive and encouraging supervisors (Shalley et al., 2000; Çekmecelioğlu, 2011; Robbins et al., 2018). Employees who are satisfied with their job can experience and display positive emotions that should be exhibited in the workplace. Therefore, supervisor support can affect emotional labor behavior in individuals through job satisfaction.

*H*6: Job satisfaction mediates the relationship between supervisor support and surface acting.

*H*7: Job satisfaction mediates the relationship between supervisor support and deep acting.

### 2.4 Autonomy, emotional labor

Hochschild (1983) discussed the discomfort arising from organizations exerting control over an individual’s personal emotional state, effectively commodifying emotions. This commercialization extends to employees who must maintain a friendly demeanor, even when dealing with challenging customers, necessitating considerable effort and resulting in emotional exhaustion and stress. The process of regulating emotions is influenced by *“personal factors”* like gender, emotional expression, emotional intelligence, and positive/negative emotions, as well as “*organizational factors”* such as job autonomy, supervisor support, coworker support, and work-related stress (Hwang and Park, 2022).

A meta-analysis focusing on emotional labor in the nursing profession found that organizational characteristics bear a stronger relationship with emotional labor compared to personal attributes (Kim and Ham, 2015). Autonomy, as defined by Hackman and Oldham (1975), pertains to the extent of an employee’s freedom, independence, and discretion in performing job duties. Employees with greater job autonomy appear to perceive emotional labor as less burdensome (Erickson, 1991; Wharton, 1993). This is primarily attributed to the fact that job autonomy alleviates stress associated with the emotional regulation process (Hochschild, 1983).

Evidence suggests that the adverse effects of emotional labor are exacerbated when individuals’ emotional responses are tightly regulated by organizational rules, while these negative consequences diminish when employees have a degree of control over their emotional reactions (Oral and Köse, 2011, 471). In an illustrative case, employees filed lawsuits against a grocery chain that mandated smiling at customers, even when subjected to sexual harassment, underscoring the detrimental consequences of organizations curtailing emotional autonomy (Grandey et al., 2007).

Wharton (1993) discovered that individuals reporting high levels of autonomy experienced lower emotional exhaustion, regardless of the type of emotional labor involved in their jobs. Similarly, a study conducted on nurses demonstrated that autonomy mitigates emotional labor (Hwang and Park, 2022). Consequently, this study posits that employees endowed with job autonomy are likely to experience more positive emotions and reduce their engagement in emotional labor. This proposition forms the basis of the following hypothesis:

*H*8: Autonomy negatively affects surface acting dimension of emotional labor.

*H*9: Autonomy positively impacts deep acting dimension of emotional labor.

Having a lack of control over events has been described as both a source of life stress (Rodin, 1986) and work stress (Grandey, 2000). Judge et al. (1997), who defines autonomy as the freedom to have control from the beginning to the end of a job or to solve problems, states that autonomy combines the interest of individuals with the goals of the organization and increases the desire to achieve. Spreitzer (1995) states that taking initiative in subjects such as starting, maintaining and controlling an activity will increase job satisfaction by making the person feel powerful. In addition, it has been observed that providing some area of work-related freedom to the employees accelerates the creative thinking process and increases the creative behaviors of the employees (Amabile et al., 1996). Morris and Feldman (1996) reported that job autonomy is positively correlated with job satisfaction and negatively related with emotional exhaustion and dissonance. There is evidence that job autonomy has a positive impact on job satisfaction and other job attitudes (Hackman and Oldham, 1975; Spreitzer, 1995; Wallin A. O. et al., 2012; Wu et al., 2018). For this reason, the following hypothesis has been proposed in line with the research findings and theoretical explanations.

*H*10: Autonomy positively affects job satisfaction.

Grandey (2000), who considers emotional labor as a psychological process that includes the management of the psychological state of employees in their interactions with customers, states that the successful management of this psychological process is achieved by the successful regulation of emotions. At this point, Grandey (2000), who defines emotional labor as the regulation of emotions and behaviors for organizational purposes through exaggeration, suppression or role playing, approaches the subject with a holistic perspective and draws attention to the concept of emotional regulation. According to this, the concept of emotional labor is related to the real feelings of the employees and the mechanisms through which they feel and reflect them. Emotional regulation is an important process in order to comply with the display rules, which are at the core of the concept of emotional labor (Beğenirbaş and Yalçın, 2012, 23). In this process, autonomy and social support play an important role as organizational factors. Giving people freedom in some areas related to their work will increase their self-confidence and will enable them to take responsibility. This, in turn, will increase a person’s satisfaction with his/her job and job conditions and enable him/her to regulate his/her emotions in a positive way in the work environment (Grandey, 2000; Grandey et al., 2007). The following hypotheses are developed in this study since it is thought that job satisfaction will act as a mediator between autonomy and emotional labor.

*H*11: Job satisfaction mediates the relationship between autonomy and surface acting.

*H*12: Job satisfaction mediates the relationship between autonomy and deep acting.

## 3 Materials and methods

### 3.1 Measures

To assess the hypotheses outlined above, we employed multi-item scales sourced from previous scholarly research to measure the various constructs under investigation. Each construct related to the next generation was evaluated using a 5-point Likert scale, with response options ranging from “strongly disagree” (1) to “strongly agree” (5). Below, we provide a concise overview of the measurement tools employed in this study.

Emotional Labor Measurement:

To quantify emotional labor, we adopted the emotional labor scale developed by Diefendorff et al. (2005), which consists of two dimensions encompassing a total of 11 items. Specifically, seven items pertain to surface acting, and four items relate to deep acting. Sample items from this scale include:

- *“I engage in a ‘show’ or ‘performance’ when interacting with patients”* (Surface acting).
- *“I strive to genuinely experience the emotions required to display to patients”* (Deep acting).

Job satisfaction measurement:

To gage job satisfaction, we utilized a five-item job satisfaction scale derived from Judge et al. (2005). Representative items from this scale include:

- *“I am reasonably content with my current job.”*
- *“I derive genuine enjoyment from my work.”*

Supervisor support measurement:

The measurement of supervisor support involved a four-item scale, and this instrument was adapted from work climate scales developed by Amabile et al. (1996), Scott and Bruce (1994), and Oldham and Cummings (1996). An example item from this scale is:

- *“My supervisor serves as a positive role model”* (Supervisor support).

Autonomy measurement:

Autonomy was assessed using a three-item scale derived from the aforementioned work climate scales. An illustrative item from this scale is:

*“I possess the freedom to determine how I will execute my tasks.”*

### 3.2 Sampling

The primary objective of this research is to elucidate and scrutinize the intricate connections among supervisor support, autonomy, job satisfaction, and emotional labor in the context of healthcare services, particularly within the nursing profession.

To empirically explore our hypotheses, by convenience sampling we selected a sample of 750 nurses working in healthcare institutions situated in Istanbul, chosen for accessibility. Initially, we reached out to all 750 nurses via phone, providing a clear explanation of the study’s objectives. Out of the contacted individuals, 448 nurses consented to participate. Subsequently, 432 of the consenting nurses completed the survey. Following a thorough review, we excluded all incomplete responses with missing data, leaving us with 416 responses for detailed analysis. The majority of the sample constituted female participants (86.8%, *n* = 361), with the largest proportion belonging to the 20–30 age group (59.1%, *n* = 246). Additionally, 56.7% of the participants were married, and the predominant educational background was bachelor’s degrees (68.5%).

In terms of their professional roles, 61% were involved in clinical units, followed by intensive care units (20.7%), outpatient clinics (9.9%), administrative units (4.8%), and the operating room (3.6%). Concerning their job titles, the largest contingent comprised clinical nurses (66.6%), followed by charge nurses and supervisors (32.9%), with a small fraction (0.5%) occupying administrative positions as deputy managers. Among the nurses, 60.4% worked on a rotating shift basis, while the remaining 39.6% adhered to regular daytime hours. Regarding patient caseloads, 34.6% handled 5–10 patients daily, 26.9% managed 30 or more patients, 17.3% attended to 11–15 patients, 11.5% were responsible for 16–20 patients, and 9.6% had 21–25 patients under their care.

Lastly, we delved into the working environment by inquiring about potential challenges within the hospital setting. Among the surveyed nurses, 69.9% acknowledged facing a substantial workload, 37.5% reported frequent difficulties in taking breaks due to the intensity of their duties, 35.6% mentioned encountering instances of violence from patients and their families, 31.7% linked disruptions in their work-life balance to night shifts, 28.6% claimed to have experienced workplace bullying, and 24.3% expressed various communication issues with their colleagues.

To conduct our analysis, we employed the Partial Least Squares Structural Equation Modeling (PLS-SEM) technique. We selected this method because it excels at modeling latent variables, handling multiple dependent constructs, and explicitly addressing measurement errors. This choice allows us to effectively test our model’s overall, direct, and indirect effects, including mediation (Hair et al., 2014). The PLS-SEM analysis in this study was conducted using SmartPLS 3.0 software. The analytical process within SmartPLS 3.0 unfolds across three distinct stages:

- *Outer model testing phase:* This phase is dedicated to scrutinizing the validity and reliability of indicators and constructs. It ensures the robustness of the measurement model.
- *Goodness of fit model testing phase:* In this stage, the model’s predictive power and overall feasibility are assessed. The examination of goodness-of-fit indicators provides insights into the model’s adequacy in explaining the observed data.
- *Inner model testing phase:* Focused on evaluating the significance of the relationships between exogenous and endogenous variables, this phase delves into the core structural aspects of the model.

Within the SmartPLS framework, the conventional significance threshold is set at *p* < 0.05, corresponding to t-statistics values exceeding 1.96 (*p* < 0.05 = T Statistics >1.96). However, more stringent significance levels of 0.01 (T > 2.576) and 0.001 (T > 3.291) are also considered acceptable, as highlighted by Novitasari (2022). These thresholds provide a nuanced evaluation of the statistical significance of the relationships under consideration.

### 3.3 Measurement validation

In this research, following the methodology of Kleijnen et al. (2007), we employed reflective indicators for all the constructs under investigation. In order to assess the psychometric properties of our measurement instruments, we initially estimated a null model devoid of structural relationships. Reliability was evaluated through various indicators, namely Composite Scale Reliability (CR), Cronbach’s alpha, and Average Variance Extracted (AVE). It is noteworthy that all these measures surpassed established thresholds. Specifically, the PLS-based CR values exceeded 0.70 for all constructs, Cronbach’s alpha values exceeded .70 as well, and the AVE values for all first-order constructs exceeded the .50 threshold.

Furthermore, we examined convergent validity by scrutinizing the standardized loadings of the measurement instruments on their respective constructs. We found that all measures exhibited standardized loadings exceeding 60.

To provide additional evidence of discriminant validity, we presented correlations among all five variables in Table 1. To meet the criteria for discriminant validity, the AVE for each construct should exceed the square of the correlation between that construct and other constructs (Fornell and Larcker, 1981). These outcomes imply that the items in our study share more common variance with their corresponding constructs than they do with other constructs (Howell and Avolio, 1993). Importantly, none of the inter-correlations among the constructs in our model exceeded the square root of the AVE of the constructs, further substantiating discriminant validity (see Table 1).

**Table 1:** Cronbach alpha, AVE, CR values and discriminant validity.

Variables	1	2	3	4	5
Autonomy	0.834				
Deep acting	0.002	0.894			
Job satisfaction	0.322	0.174	0.844		
Supervisor support	0.302	0.118	0.455	0.909	
Surface acting	−0.161	0.253	−0.186	−0.088	0.767
CR	0.872	0.923	0.925	0.950	0.876
AVE	0.695	0.800	0.713	0.827	0.588
α	0.786	0.879	0.898	0.930	0.828

In order to estimate the indirect effects, main effects, and test the hypotheses, we employed the PLS (Partial Least Squares) approach as outlined by Chin et al. (2003). We utilized the bootstrapping resampling method and conducted these computations using the SmartPLS 3.0 software program. To establish the statistical significance of the links, we computed T-statistics for all coefficients and ensured their stability across sub-samples.

The path coefficients, along with their associated t-values, provided insights into the direction and magnitude of each hypothesized relationship (Hackman et al., 1975). The results of our hypotheses, including the paths, beta coefficients, and significance levels, are presented in Table 2.

**Table 2:** Path results.

Relationships			Path coefficient (β)	Hypotheses	Results
Supervisory support	→	Surface acting	.009	H1	Not supported
Supervisory support	→	Deep acting	0.068	H2	Not Supported
Supervisory support	→	Job satisfaction	0.395**	H3	Supported
Job satisfaction	→	Surface acting	−0.134*	H4	Supported
Job satisfaction	→	Deep acting	0. 162*	H5	Supported
Autonomy	→	Surface acting	−0.142*	H8	Supported
Autonomy	→	Deep acting	−0.074	H9	Not Supported
Autonomy	→	Job satisfaction	0.201**	H10	Supported

*p < 0.05. **p < 0.01.

Our findings indicate that both supervisory support (*β* = 0.395; *p* < 0.01) and autonomy (*β* = 0.201; *p* < 0.01) exert positive and statistically significant effects on job satisfaction, thus providing support for H3 and H10. Additionally, job satisfaction displays a statistically significant negative relationship with surface acting (*β* = −0.134; *p* < 0.05) but a positive and significant association with deep acting (*β* = 0.162; *p* < 0.05), corroborating H4 and H5. Moreover, autonomy demonstrates a statistically significant negative relationship with surface acting (*β* = −0.142; *p* < 0.05), aligning with H8.

However, our findings do not offer empirical support for direct relationships between supervisory support and any emotional labor dimensions, nor do they establish a direct link between autonomy and deep acting. Consequently, H1, H2, and H9 are not substantiated by the empirical evidence.

Furthermore, we conducted mediation analyses to examine the potential mediating role of job satisfaction in the connections between supervisory support and emotional labor (specifically surface acting and deep acting) and between autonomy and emotional labor (again, specifically surface acting and deep acting). Notably, the total effects analysis revealed statistically significant associations between supervisory support and deep acting, as well as between autonomy and surface acting. Consequently, we focused our mediation examination exclusively on these specific paths, excluding the others.

Upon introducing job satisfaction as a mediating variable, the impact of supervisory support on deep acting (*β* = 0.068; *p* > 0.05) lost its statistical significance. In the case of autonomy and surface acting, the association remained significant (*β* = −0.125; *p* < 0.05) but with a somewhat reduced magnitude. However, the indirect effects were notably significant: (i) for supervisory support on deep acting (*β* = 0.064; *p* < 0.05), and (ii) for autonomy on surface acting (*β* = −0.027; *p* < 0.05). These findings suggest that the relationship between supervisory support and deep acting is entirely mediated by job satisfaction, while the relationship between autonomy and surface acting is only partially mediated. As a result, our hypotheses H7 and H11 receive empirical support (Table 3).

**Table 3:** Results for the mediating analyses.

Total effect		Direct effect		Indirect effect			
Relationship	Path coefficient (β)	Relationship	Path coefficient (β)	Relationship	Path coefficient (β)	BI[2.5%;97.5%]	
SS→SA	−0.044	SS→SA	0.009	SS→JS→SA	−0.053	−0.102	0.015
SS→DA	0.132**	SS→DA	0.068	SS→ JS→DA	0.0 64*	0.012	0.120
Au→SA	−0.169*	Au→SA	−.142*	Au→ JS→SA	−.027*	0.059	0.005
Au→DA	−0. 042	Au→DA	−0.074	Au→ JS→DA	0.033	−0.007	0.069

SS, Supervisory support; Au, Autonomy; SA, Surface acting; DA, Deep acting.*p < 0.05. **p < 0.01.

### 3.4 Structural model

To validate the effectiveness of the PLS-SEM approach, we assessed various quality indicators, including the coefficient of determination (R^2^; Chin et al., 2003), Q predictive validity (Q2), Normed Fit Index (NFI), and Standardized Root Mean Squared Residual (SRMR; Tenenhaus et al., 2005).

The R^2^ values of the endogenous constructs were employed to evaluate model fit, signifying how well data points align with a line or curve (Chin et al., 2003; Tenenhaus et al., 2005). Following Chin’s recommendations, we categorized R^2^ values as small (0.02 ≤ R^2^ < 0.13), medium (0.13 ≤ R^2^ < 0.26), or large (0.26 ≤ R^2^). Our analysis reveals that individual creativity (R^2^ = 0.244) demonstrates a medium effect size, while surface acting (R^2^ = 0.049) and deep acting (R^2^ = 0.36) exhibit small effect sizes (Table 4). Additionally, we found that the Q predictive validity for all our endogenous constructs met the criteria, indicating that the predictors within the model can account for variance in the dependent variable.

**Table 4:** Structural model.

Endogenous constructs	R^2^	Q2	SRMR	NFI
Job satisfaction	0.244	0.224	0.064	0.839
Surface acting	0.049	0.085		
Deep acting	0.036	0.072		

In terms of model fit criteria for PLS-SEM, we considered two key metrics: the Standardized Root Mean Squared Residual (SRMR) and the Normed Fit Index (NFI). It is recommended that the SRMR value should be equal to or less than 0.08 (Hu and Bentler, 1998), and the NFI value should exceed 0.90 (Hair et al., 2017). Our model aligns with these criteria as evidenced by the SRMR value of 0.070, which satisfies the recommendation, and the NFI value of 0.839, which approaches the desired threshold. Consequently, we can confidently conclude that the developed structural model exhibits strong predictive power and is highly satisfactory.

Moreover, we assessed the minimum sample size requirement through the minimum R-squared method, a technique advocated by Hair et al. (2014, p. 21) in their seminal work on Partial Least Squares Structural Equation Modeling (PLS-SEM). This method, based on Cohen (1988, 1992) power tables for least squares regression, utilizes a table that outlines the minimum required sample sizes determined by three key factors. The first factor in the minimum R-squared method is the maximum number of arrows directed at a latent variable (also known as a construct) within a model. The second factor is the chosen significance level, with the commonly adopted level of 0.05 leading to a corresponding power level of 0.8. The third factor is the minimum R-squared value within the model. For our model, the minimum R-squared is 0.036, and the maximum number of arrows (paths) directed at a latent variable is 3. To estimate the minimum sample size required, we utilized Table 5, adapted from Kock and Hadaya (2018), as a condensed version of the original table presented by Hair et al. (2014, p. 21). According to our calculations, the minimum sample size necessary for our model is 124. Consequently, we deem our sample, consisting of 416 nurses, to be adequate.

**Table 5:** Table for the minimum R-squared method.

Maximum number of arrows pointing at a construct	Minimum R^2^ in the model			
	0.10	0.25	0.50	0.75
2	110	52	33	26
3	124	59	38	30
4	137	65	42	33
5	147	70	45	36
6	157	75	48	39
7	166	80	51	41
8	174	84	54	44
9	181	88	57	46
10	189	91	59	48

## 4 Results and discussion

Numerous international studies consistently underscore the challenging work environment faced by nurses, which is associated with professional burnout, diminished job satisfaction, workplace conflicts, increased emotional demands, incidents of workplace violence, and heightened work-related stress (Bakker et al., 2004; Aiken et al., 2009; Nantsupawat et al., 2011; Wu et al., 2018). Unfortunately, research conducted in Turkey mirrors these findings, highlighting that Turkish nurses encounter analogous challenges in their professional milieu. The outcomes of our study corroborate the prevailing notion that nurses grapple with multifaceted challenges in their work environment. These challenges encompass prolonged working hours and night shifts, primarily driven by staffing shortages.

It is noteworthy that our study sample predominantly consists of young female nurses, signifying a demographic that may be particularly susceptible to emotional labor challenges due to their central roles in patient care. The marital status of these nurses underscores the importance of work-life balance within the nursing profession, an aspect closely linked to emotional well-being. Furthermore, the prevalence of bachelor’s degrees among the participants suggests an expectation of heightened professional autonomy. The diversity of clinical settings where nurses work emphasizes the multifaceted nature of nursing, necessitating a wide spectrum of emotional labor and managerial skills. Professional titles within the nursing profession reveal a hierarchical structure that can significantly influence emotional labor and autonomy. Variances in work schedules and patient caseloads have a substantial impact on nurses’ emotional experiences, job satisfaction, and their perception of autonomy. Consequently, the nursing work environment is characterized by intricate dynamics, including stress related to hierarchy, challenges in patient care, and potential reductions in job satisfaction and organizational commitment. These issues are often attributed to factors such as work-life balance and emotional exhaustion (Morris and Feldman, 1996; Brotheridge and Grandey, 2002; Aiken et al., 2011; Kim et al., 2017).

One of the foremost issues encountered by nurses, both globally and in Turkey, is workplace violence within the healthcare sector (Günaydin and Kutlu, 2012; Kaya and Turan, 2016; Jafree, 2017; Berlanda et al., 2019; Akbolat et al., 2021; Yıldız and Yıldız, 2022). Workplace violence in healthcare encompasses verbal abuse, threatening behavior, or physical assault directed at healthcare workers by patients, patients’ relatives, or members of the community (Saines, 1999). Notably, Turkish healthcare personnel, particularly nurses, experience a high prevalence of violence, with mobbing affecting 17.1% of the nursing workforce (Aksakal et al., 2015, 1361). This trend is not unique to Turkey but is recognized as a global phenomenon, with international organizations such as the World Health Organization highlighting the vulnerability of healthcare workers, particularly nurses, to social and political violence in disaster, conflict, and war settings (World Health Organization, 2021). Regrettably, exposure to violence in the healthcare sector contributes to reduced job satisfaction among employees (Hershcovis and Barling, 2009).

Studies conducted on Turkish nurses consistently reveal elevated levels of emotional labor, with nurses initially engaging in more surface acting behaviors early in their careers, transitioning toward deeper emotional acting behaviors in later years (Doğan and Sığrı, 2017; Aydın and Kalemci Tüzün, 2019). Additionally, some studies have reported that nurses predominantly exhibit sincere behavior, with lower levels of surface acting behavior (Demir et al., 2021). Conversely, Tunç et al. (2014) concluded that no significant difference was found in emotional labor strategies between nurses working in different departments, indicating similar levels of surface acting behavior, emotional effort, and deep acting behavior among intensive care and inpatient service nurses. Değirmenci Öz and Baykal (2018), in their examination of factors affecting nurses’ emotional labor behavior, noted that nurses in private hospitals tend to suppress their emotions more. Those who voluntarily choose nursing as a profession tend to suppress their emotions less, while those working daytime shifts and who have chosen the profession willingly are more likely to exhibit deep acting behavior. These disparate findings underscore the complexity of emotional labor within nursing, with results varying across studies.

In a study encompassing 11,000 nurses across 92 countries, Wu et al. (2018) found low job satisfaction among nurses, particularly concerning wages and work-family balance. High levels of deep acting and low levels of surface acting were also observed. Karimi et al. (2014) investigated emotional labor, emotional intelligence, and work stress among nurses, concluding that nurses with elevated emotional labor levels experienced lower levels of well-being and heightened work stress.

Hospital administrators view emotional labor done by nurses as a crucial component of their jobs that must be met in order to enhance job performance, including customer-oriented care and service quality.

Furthermore, research indicates that many nurses working in clinical settings grapple with burnout due to the substantial emotional energy required to balance patient needs while simultaneously maximizing hospital profits, which can lead to reduced job performance. Interestingly, hospital administrators perceive emotional labor done by nurses as a crucial component of their jobs that must be met in order to enhance job performance, including customer-oriented care and service quality (Kim et al., 2009). Indeed, emotional labor is deemed an organizational necessity, not merely for meeting patient needs but also for ensuring hospital profitability and efficiency (Hwang and Park, 2022). However, employees’ emotional labor may not manifest spontaneously, with various individual and organizational factors influencing this phenomenon. Some studies have suggested that organizational characteristics exert a more substantial influence on emotional labor than personal attributes (Kim and Ham, 2015). Numerous studies emphasize the significance of the work environment in shaping positive job attitudes among service sector employees, especially in healthcare settings. The work environment significantly affects employees’ emotional labor displays (Ashforth and Humphrey, 1993; Morris and Feldman, 1996; Grandey, 2000; Brotheridge and Grandey, 2002; Aiken et al., 2011; Kim et al., 2017).

In our study, which explores the effect of supervisor support and autonomy dimensions within the work environment on job satisfaction and emotional labor, the results demonstrate that supportive managerial behaviors enhance employee job satisfaction significantly. This result highlights the crucial role of supervisor support, particularly in ameliorating the job satisfaction of nurses who operate under challenging conditions in healthcare settings. In this context, supervisors who motivate their employees through verbal encouragement, constructive feedback, problem-solving facilitation, and open communication channels are crucial in boosting nurses’ job satisfaction. Other studies have similarly highlighted that enhancing the organizational climate can augment nurses’ job satisfaction (Wallin L. et al., 2012; Wu et al., 2018). Likewise, assertiveness supported by management, coupled with organizational and team support, can elevate job satisfaction and organizational commitment while decreasing turnover intentions (Çekmecelioğlu, 2005; Çekmecelioğlu and Günsel, 2011).

In our study, we did not obtain statistically significant results concerning the impact of supervisor support on emotional labor. This outcome may be attributed to the emotional exhaustion experienced by nurses working under adverse conditions, exacerbating the stress they encounter. Other studies focusing on the antecedents of emotional labor among service sector employees in Turkey have yielded results indicating that supportive managerial behaviors and equitable, transparent conduct by supervisors significantly influence employee emotional labor behaviors (Beğenirbaş and Yalçın, 2012; Cinnioğlu and Salha, 2017; Kafadar and Kaygın, 2017; Kavak and Kaygın, 2018; Çekmecelioğlu et al., 2021) have shown that supportive managerial behaviors and equitable, transparent conduct by supervisors significantly influence employee emotional labor behaviors.

Our research also explores the relationship between job satisfaction and emotional labor from a novel perspective. It suggests that job satisfaction can be regarded a precursor to emotional labor. The theoretical foundation of this idea is the Job Demands-Resources (JD-R) model put forth by Demerouti et al. (2001). This model examines factors related to the well-being and stress of workers, emphasizing the balance between job demands and job resources as a key determinant. According to this model, job satisfaction can be seen as a result of the equilibrium between job resources and job demands. Research findings indicate that job satisfaction affects both surface acting and deep acting emotional labor behaviors.

Job satisfaction has a diminishing impact on surface acting, characterized by the display of emotions that individuals do not genuinely feel, thus reducing the occurrence of this inauthentic behavior. In contrast, it positively influences deep acting emotional labor, in which individuals try to genuinely experience and express emotions by empathizing with others. Therefore, our study underscores the significance of job satisfaction as a precursor to emotional labor.

One particularly noteworthy finding is the mediating role played by job satisfaction in these relationships. Specifically, job satisfaction acts as a complete mediator between supervisory support and deep acting. In other words, when nurses perceive strong support from their supervisors, their job satisfaction increases, subsequently facilitating their engagement in deep acting emotional labor, characterized by the genuine expression of emotions. This underscores the pivotal role of supportive leadership in fostering a positive work environment, influencing how nurses manage their emotional labor.

Furthermore, our study reveals that job satisfaction partially mediates the connection between autonomy and surface acting. Nurses who have greater autonomy in their roles tend to lead to higher job satisfaction, which, in turn, somewhat diminishes their inclination to engage in surface acting emotional labor—in which they express emotions that do not align with their genuine feelings. This emphasizes the significance of empowering nurses with job autonomy, as it contributes to their job satisfaction and alleviates their need to display inauthentic emotions.

In conclusion, as the service sector grows and competition intensifies, organizations have placed increasing emphasis on the customer service quality. Given that perceived service quality is heavily influenced by customer interactions with service providers and their employees, how service providers communicate with customers has become a critical concern for organizational management. This holds especially true in the healthcare sector, where emotional demands on employees, particularly nurses, are exceptionally high. Nurses often face heavy workloads, long hours, night shifts, and stressful working conditions, which can adversely affect their job satisfaction and their ability to manage emotional labor. Specifically, our findings underscore the importance of a supportive work environment and autonomy in influencing nurses’ job satisfaction and emotional labor. This aligns with the identified gap in understanding the organizational factors shaping emotional labor in healthcare settings.

Our research highlights the critical role of the work environment, specifically supervisory support and autonomy, in enhancing job satisfaction among nurses. This, in turn, positively affects their emotional labor behaviors. It suggests that organizational leaders and managers should focus on fostering a supportive work environment and providing nurses with the autonomy to make decisions, solve problems, and take initiative. Such measures can help reduce the emotional demands on nurses and improve the overall work environment, ultimately leading to increased job satisfaction and better emotional labor management. Moreover, organizational leaders and managers should also consider how organizations, particularly healthcare institutions, can implement strategies to enhance supervisory support and autonomy for nurses. Training programs for supervisors to improve supportive behaviors and initiatives to empower nurses with greater job autonomy may be helpful.

### 4.1 Limitations and future directions

Addressing concerns regarding the generalizability of our study is paramount for a comprehensive understanding of its implications. Our research concentrated exclusively on nurses within public hospitals in a specific region of Turkey, providing valuable insights into the contextual dynamics of emotional labor. However, caution is warranted when extending these findings to nurses in private hospitals or different healthcare settings. To enhance the external validity of our results, future research endeavors should prioritize the inclusion of a more diverse sample, encompassing nurses from both public and private healthcare sectors.

Furthermore, our study’s focus on nurses as a distinct professional group raises important considerations about the transferability of our findings to other occupational sectors. While our insights contribute significantly to the understanding of emotional labor among nurses, the application of these findings to different professions may require careful scrutiny. To address this limitation, future research should explore emotional labor across various occupational groups, facilitating a comparative analysis of emotional labor levels and determinants. This approach would enable a more nuanced comprehension of the unique emotional labor challenges faced by professionals in different fields.

Another noteworthy limitation pertains to the specific demographic and organizational context of our study, as it was exclusively conducted on nurses in public hospitals within a particular region of Turkey. Recognizing this constraint, future investigations should delve into the work environments and emotional labor behaviors of nurses in private hospitals. A comparative exploration between public and private healthcare. In addition to supervisory support and autonomy, other aspects of the organizational climate, such as job quality and teamwork, could be further investigated to determine their effects on emotional labor among healthcare professionals.

Since our study primarily is focused on supervisory support and autonomy, future research should delve into other aspects of the organizational climate. Factors such as job quality, teamwork, and organizational culture may exert additional influences on emotional labor among healthcare professionals. Investigating these elements can provide a more comprehensive understanding of the organizational determinants of emotional labor. Moreover, future research should also explore whether positive job attitudes, like organizational commitment, may be precursors to emotional labor; future studies should explore these relationships. Understanding how positive perceptions of the organization contribute to emotional labor behaviors can shed light on the motivational aspects influencing healthcare professionals.

Furthermore, our investigation did not include an assessment of potentially influential protective factors, such as psychotherapy and counseling interventions, which individuals often utilize without prescription to mitigate symptoms. This omission represents a limitation in comprehensively capturing all potential factors influencing emotional labor within healthcare settings. Exploring how individuals utilize such interventions to cope with the emotional demands of their profession can contribute to a more nuanced understanding of emotional labor within healthcare settings.

Finally, as emotional labor has predominantly been the subject of research within the service sector, there is an evident need for expanded exploration into its dynamics across various industries. Despite limited endeavors, some studies have initiated the exploration of emotional labor beyond the service sector, indicating the potential for broader investigations. Future research endeavors are encouraged to extend their scope beyond the service sector, delving into different professional contexts. This broader exploration can contribute to a more comprehensive understanding of emotional labor, shedding light on both universal principles and context-specific nuances that may vary across diverse industries. By venturing beyond the confines of the service sector, researchers can uncover new insights into the multifaceted nature of emotional labor, enriching our knowledge and enhancing the applicability of findings across a range of professional domains.

## Data availability statement

The raw data supporting the conclusions of this article will be made available by the authors, without undue reservation.

## Ethics statement

The studies involving humans were approved by Sakarya University Social and Humanities Ethical Board. The studies were conducted in accordance with the local legislation and institutional requirements. The participants provided their written informed consent to participate in this study.

## Author contributions

GK: Conceptualization, Data curation, Formal analysis, Investigation, Methodology, Project administration, Resources, Software, Supervision, Validation, Writing – original draft, Writing – review & editing. SA: Conceptualization, Data curation, Formal analysis, Investigation, Methodology, Project administration, Resources, Software, Supervision, Validation, Writing – original draft, Writing – review & editing. HG: Conceptualization, Data curation, Formal analysis, Investigation, Methodology, Project administration, Resources, Software, Supervision, Validation, Writing – original draft, Writing – review & editing. MG: Conceptualization, Data curation, Formal analysis, Investigation, Methodology, Project administration, Resources, Software, Supervision, Validation, Writing – original draft, Writing – review & editing.
